# Supplementary material for: Inhibition of E. coli RecQ Helicase Activity by Structurally Distinct DNA Lesions: Structure—Function Relationships
Source: Int J Mol Sci. 2022 Dec 9;23(24):15654. doi: 10.3390/ijms232415654 (PMC9779537; doi:10.3390/ijms232415654)
Supplement: Supplementary file 1 [file ijms-23-15654-s001.zip › ijms-1975751-supplementary.pdf]

Supporting Materials

Inhibition of *E. coli* RecQ Helicase Activity by Structurally Distinct DNA Lesions: Structure – Function Relationships.

Ana H. Sales, Vincent Zheng, Maya A. Kenawy, Mark Kakembo, Lu Zhang[#], Vladimir Shafirovich, Suse Broyde[*] and Nicholas E. Geacintov

Author correspondence: Nicholas E. Geacintov, ng1@nyu.edu.

1. The following experiment demonstrates that during the course of the experiments described in the body of the manuscript, the reannealing of the unwound BHQ fluorescence quencher with the C3-strand is unlikely at the 5 nM DNA concentration used in our experiments.

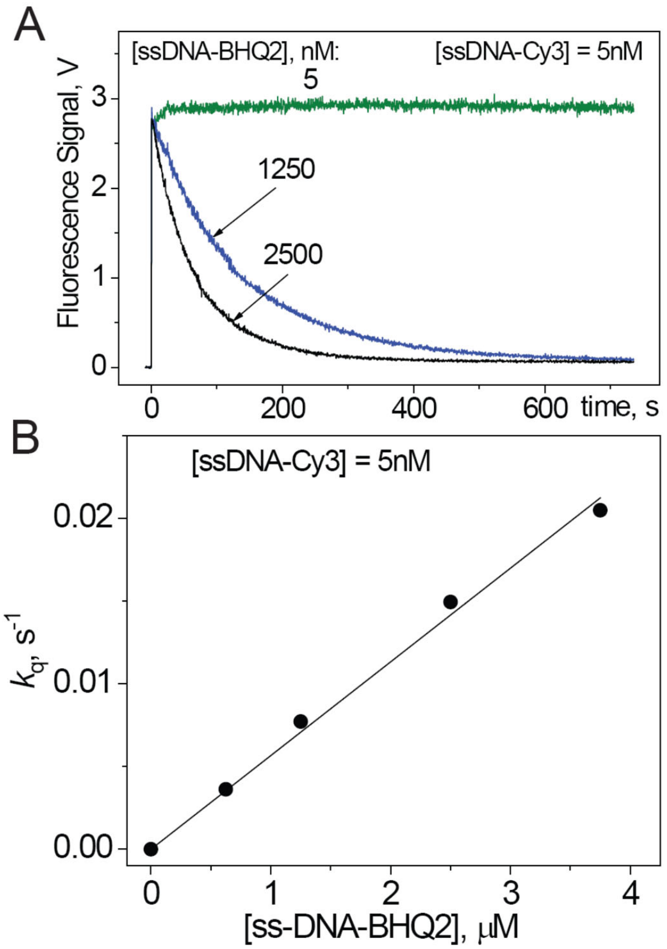

Figure S1. (**A**) Kinetics of fluorescence quenching initiated by the addition of 47-mer single-stranded oligonucleotides containing a BHQ2 quencher at the 3′-end (ssDNA-BHQ2) to 32-mer single-stranded oligonucleotides (5 nM) labelled with the Cy3 dye at the 5′-end (ssDNA-Cy3). No quenching observed at the 5 nM strand concentrations. (**B**) Dependence of the first-order rate constant of fluorescence quenching (k_q) on the concentration of the quencher oligonucleotide (ssDNA-BHQ2). The solid line shows the best linear fit of the bimolecular rate constant of fluorescence quenching (k_Q) to the data points at $k_Q = (5.7\pm0.2)\times10^3\ M^{-1}s^{-1}$. Consistent with panel (A), the bimolecular quenching constant is too small to be effective in the nM concentration range.

These results demonstrate that there is no re-annealing at the 5 nM DNA concentration used in our experiments (green line on the top). However, quenching does occur at higher concentrations of the BHQ strand.

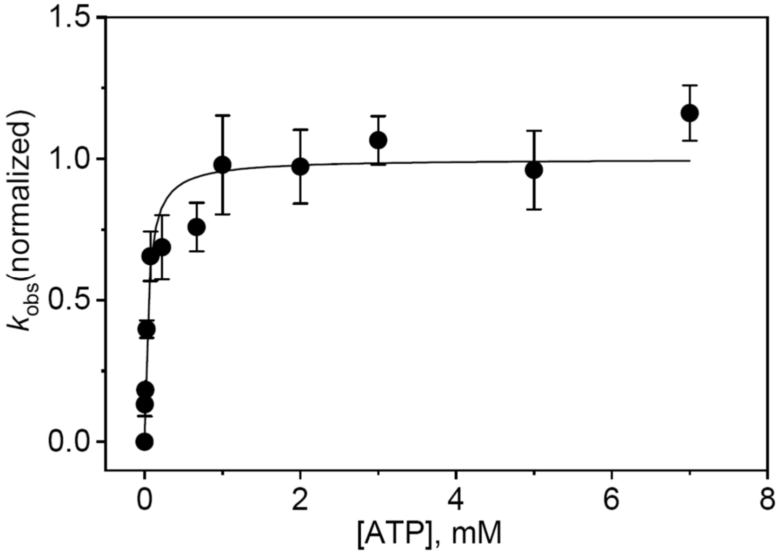

Figure S2. Effect of ATP concentration on the observed reaction rate constant. The values of k_{obs} obtained at different ATP concentrations were normalized relative to the $k_{obs} = 0.0049$ s^{-1} value measured at [ATP] = 2 mM, [DNA] = 5 nM.
